# The aging mouse microbiome has obesogenic characteristics

**DOI:** 10.1186/s13073-020-00784-9

**Published:** 2020-10-12

**Authors:** Dana Binyamin, Nir Werbner, Meital Nuriel-Ohayon, Atara Uzan, Hadar Mor, Atallah Abbas, Oren Ziv, Raffaele Teperino, Roee Gutman, Omry Koren

**Affiliations:** 1 Azrieli Faculty of Medicine, Bar-Ilan University, Safed, Israel; 2 Laboratory of Integrative Physiology, MIGAL - Galilee Research Institute, Kiryat Shmona, Israel; 3 Institute of Experimental Genetics, Helmholtz Zentrum München, German Research Center for Environmental Health, Neuherberg, Germany; 4 German Center for Diabetes Research (DZD), Neuherberg, Germany; 5 Department of Animal Sciences, Faculty of Sciences and Technology, Tel-Hai College, 12210 Upper Galilee, Israel

**Keywords:** Aging, Microbiome, Metabolism, Fecal microbiota transplantation

## Abstract

**Background:**

During aging, there is a physiological decline, an increase of morbidity and mortality, and a natural change in the gut microbiome. In this study, we investigated the influence of the gut microbiome on different metabolic parameters in adult and aged mice.

**Methods:**

Fecal and blood samples from adult (*n* = 42, 100–300 days) and aging (*n* = 32, 550–750 days) mice were collected. Microbiome analysis was done using QIIME2. Mouse weight and body composition were measured using NMR, and insulin and leptin levels in the blood were measured with Mouse Adipokine Magnetic Bead Panel kit. Fecal microbiota transplantation experiments from adult and aged mice into young germ-free mice were carried out in order to examine the effect of the gut microbiome of adult and aging mice on weight, body composition, insulin, and leptin.

**Results:**

We demonstrate that the microbiomes from adult and aged mice are distinguishable. We also report changes in metabolic parameters as we observed significantly higher weight and fat mass and low lean mass in aged compared to adult mice along with high insulin and leptin levels in the blood. The transplanted gut microbiome from aged mice transferred part of the phenotypes seen in aged mice. Fat body mass and insulin levels were higher in the mice who received feces from aged mice than mice receiving feces from adult mice. In addition, they consumed more food and had a higher respiratory quotient compared to mice receiving adult feces.

**Conclusions:**

We conclude that aged mice have a gut microbiota with obesogenic characteristics. In addition, the gut bacterial population itself is sufficient to induce some of the manifestations of obesity.

## Background

Aging is a time-dependent multifactorial process, involving changes in various physiological systems, and complex interactions of genetic, epigenetic, and environmental factors [1, 2]. Over time, most organisms experience a physiological decline and increased morbidity and mortality due to various diseases [3]. As human lifespan is increasing worldwide, the morbidity and functional decline associated with aging have become an even more serious public health concern. Securing sufficient resources to care for an increasing elderly population, suffering from multiple diseases, is becoming a severe socioeconomic problem [4]. Reaching old age in good health is one of the greatest challenges of our society.

The gut microbiome, the collection of microorganisms residing in the digestive system and co-existing with the host, has an important role in human health and disease. The microbiome is established shortly after birth and stabilizes during the first 2–3 years of life [5]. Natural changes in the intestinal bacterial population occur during aging, due to a reduction or alteration in many physiological processes and lifestyle changes, particularly diet, metabolism, energy homeostasis, and immunity [6]. Due to the increased incidence of chronic disease, elderly individuals also experience more frequent hospitalizations, chronic infections, and extensive use of medications, including antibiotics [7], which also lead to microbiome alteration. The human gut microbiota of older people differs from that of younger adults and has the characteristics of a perturbed microbiome. These characteristics include a decrease in bacterial diversity, higher abundance of pathobionts, and a decrease in health-promoting bacteria [1].

Aging and microbiome alterations have also been investigated in mice. A distinct series of microbiome changes has been found in aged mice, which partially resembles the changes observed in humans [6]. Matsumoto et al. reported that probiotic treatment can increase longevity in mice, possibly through suppression of chronic low-grade inflammatory processes [8]. Another approach for inducing changes in the bacterial composition in the gut is by fecal microbiota transplantation (FMT). A recent study performing FMT from young and aged mice into young germ-free (GF) mice reported differences in hippocampal neurogenesis, intestinal growth, and butyrate-producing bacteria [9].

Gut microbiome research in humans is complicated by the difficulty of finding individuals in whom aging is not accompanied by disease or other environmental factors that can impact the gut microbiota composition, such as medication, decreased physical activity, or changes in diet. The relatively short lifespan of mice, the uniformity of their diet under laboratory conditions, and the ability to manipulate their microbiome make them a useful model system to study interactions between “healthy” aging and the microbiome.

In this study, we investigated the differences in the mouse gut microbiome between adult and aged mice. We also examined the influence of aging on weight, body fat mass, and insulin and leptin in the blood in mice. These experiments were designed to test the phenotypes that occur in adult and aged mice, in order to assess if these phenotypes can be transmitted to germ-free mice by fecal microbiota transplantation. We performed FMT from adult and old mice into young GF mice. Here we focused on metabolic differences and show that the gut microbiome of aged mice is different from that of adult mice and has obesogenic properties upon transfer.

## Methods

### Mice

The study was performed on FVB/N and GF Swiss-Webster mice, according to ethics approval number IL-16-01-27 and 75-11-2016. FVB/N mice were housed in the same room, under specific pathogen-free (SPF) conditions with a 12 h light/dark cycle and maintained at 22 °C ± 1 at the animal facility of MIGAL - Galilee Research Institute. GF Swiss-Webster mice were housed in isolated cages and maintained on a 12 h light/dark cycle at 22 ± 1 °C at the animal facility of the Azrieli Faculty of Medicine, Bar-Ilan University.

### Sample collection

Fecal samples were collected from male and female mice of different ages and immediately frozen at − 30 °C. The FVB/N WT mice were sampled at two-time points: adult (*n* = 42, 100–300 days) and aged (*n* = 32, 550–750 days). After 4 h of fasting, blood was collected from the submandibular vein, using Goldenrod lancets (MEDIpoint, Mineola, NY, USA) into lithium heparin-coated tubes (Greiner, Kremsmünster, Austria), which were immediately placed on ice. Tubes were then centrifuged at 1500*g* for 15 min at 4 °C. Plasma was then transferred to a clean tube and stored at − 80 °C.

### DNA extraction, amplification, and sequencing from mice feces

Total DNA was extracted from fecal samples using the PowerSoil DNA Isolation Kit (MoBio, Carlsbad, USA), according to the manufacturer’s protocol, and following a 2-min bead-beating step (Biospec). The V4 region of the bacterial 16S rRNA gene was amplified using the 515F and 806R barcoded primers following the Earth Microbiome Project protocol [10]. PCR protocol included 2 μl 515F primer (10 μM), 2 μl 806R primer (10 μM), 25 μl PrimeSTAR Max PCR Readymix (Takara, Mountain View, USA), 17 μl ultra-pure water, and approximately 20 ng of DNA. PCR reaction conditions included 3 min at 95 °C followed by 30 cycles of [10 s at 98 °C, 5 s at 55 °C, and 20 s at 72 °C], and final elongation for 1 min at 72 °C. Amplicons were purified using AMPure XP magnetic beads (Beckman Coulter, FL, USA) according to the manufacturer’s protocol. DNA was quantified using Quant-iT PicoGreen dsDNA Assay Kit (Invitrogen, Carlsbad, USA), and equimolar amounts of DNA were pooled from each sample, to ensure equal read depth. After running on a 2% agarose E-Gel (Invitrogen, Carlsbad, USA), DNA was extracted from the gel using NucleoSpin Gel and PCR Clean-up (Macherey-Nagel, Düren, Germany) and sequenced using the Illumina Miseq platform at the Genomic Center, Azrieli Faculty of Medicine, BIU, Israel.

### Bioinformatic analysis

Sequence reads were demultiplexed using QIIME2 software [11], and errors were corrected by DADA2 [12]. A phylogenetic tree was generated, and taxonomy was classified using the Greengenes reference database at a confidence threshold of 99% [13]. Alpha and beta diversity analyses were performed based on a feature table with samples containing at least 11,000 sequences. For beta diversity, Principal Coordinate Analysis (PCoA) was performed using unweighted UniFrac distances [14]. Faith’s Phylogenetic Diversity [15] was used to calculate alpha diversity. In order to identify differentially abundant taxa, ANCOM was used [16].

### Blood sample analysis

Insulin and leptin levels were measured using Mouse Adipokine Magnetic Bead Panel kit (Millipore Corporation, Billerica, MA, USA) according to the manufacturer’s protocol. Measurements were performed using the Bio-Plex MAGPIX Multiplex Reader (Bio-Rad Laboratories, Hercules, CA, USA).

### Fecal microbiome transplantation (FMT)

Fecal pellets from the mice study group (adult and aged) were kept in − 80 C° until used. Fecal samples from 8 adult and 5 aged mice were selected randomly and each pellet was suspended in 5 ml sterile PBS. After allowing large particles to settle, 200 μl of the supernatant was transferred through oral gavage into 8-week-old GF male Swiss Webster mice (*n* = 13).

### Metabolic phenotyping of GF male Swiss Webster mice following FMT

After FMT, mice were transferred to MIGAL-Galilee Research Institute, and their metabolic phenotype including body weight gain, body composition, total 24-h food (energy) intake, total 24-h energy expenditure, and respiratory quotient was measured over the following 10 days [17]. Indirect calorimetry measurements were performed using an 8-cage multi-plex system equipped with food hoppers connected to load cells for food intake monitoring (Promethion Metabolic Cage System, Sable Systems, Las Vegas). Mice were placed with bedding in the calorimetry unit for 10 days, and measurements were recorded after a 2-day habituation [17]. Raw data were collected for each mouse for 0.5 min periods, at 5-min intervals and processed by ExpeData v.1.8.2 (Sable Systems), including all steps of data correction and transformation. The system sampled CO_2_, O_2_, and H_2_O levels in the cage air, and food consumption was monitored by weight of the food dispenser. Respiratory quotient (RQ) was calculated as the volume of carbon dioxide released, and the volume of oxygen absorbed during respiration.

### Body composition

Body composition [fat mass (FM), fat-free mass (FFM), and extracellular fluid (fluids)] was measured on days 0 and 10 using Nuclear Magnetic Resonance (NMR; Minispec LF50, Bruker Optics, Germany), as previously reported [17].

### Statistical analysis

Differences between unweighted UniFrac distances were analyzed using a pairwise Permanova test. For comparison between the groups for Firmicutes to Bacteroidetes ratio, weight, fat-mass, lean-mass (fat-free mass), and insulin and leptin levels, paired two-tailed *T* tests were performed. Correlation of bacteria abundance with weight and fat/lean percent mass was done using Calour [18] and was calculated using Spearman’s statistical method and dsFDR corrections for multiple comparisons. For FMT results, an unpaired one-tailed *T* test was used, and for food consumption and average RQ, unpaired two-tailed *T* tests were done, *p < 0.05, **p < 0.01 and ***p < 0.001.

## Results

### Differences in the gut microbiome diversity of adult and aged mice

**Fig. 1:**
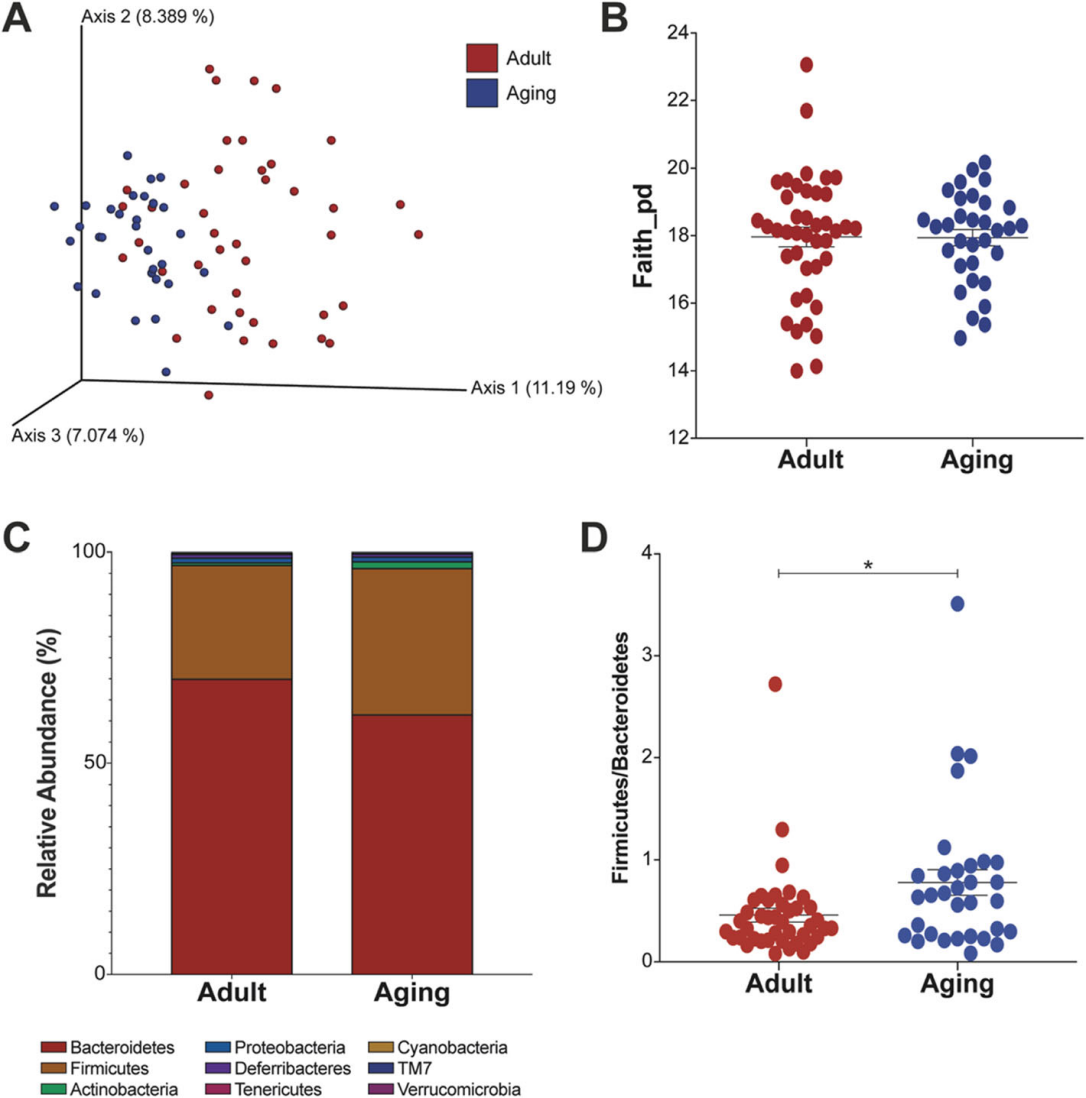
Microbial diversity differences between adult and aged mice. Fecal pellets were sampled from adult (*n* = 42) and aging (*n* = 32) mice. **a** Beta-diversity using Principal Coordinate Analysis (PCoA) of unweighted UniFrac distances (*p* = 0.001). **b** Alpha-diversity using Faith’s Phylogenetic Diversity. **c** Taxonomy plot at the phylum level. **d** Firmicutes to Bacteroidetes ratio (*p = 0.024)

To examine the changes in the microbiome during aging, we collected fecal samples from adult and aged mice. We found significant differences in beta-diversity between samples taken from adult versus aged mice (*p* = 0.001; Fig. 1a). The samples from adult mice exhibited greater β-diversity compared to the aged mice, which clustered together, indicating that the microbiomes of aged mice are more similar to one another. We did not find significant differences in bacterial richness between the age groups (Fig. 1b). When examining the relative bacterial abundance, 9 phyla were detected (Fig. 1c). Bacteroidetes tended to be lower in aged mice, while Firmicutes and Actinobacteria tended to be higher, compared with the adult animals. The Firmicutes to Bacteroidetes ratio (Fig. 1d) was significantly higher in the aged mice (*p* = 0.024).

**Fig. 2:**
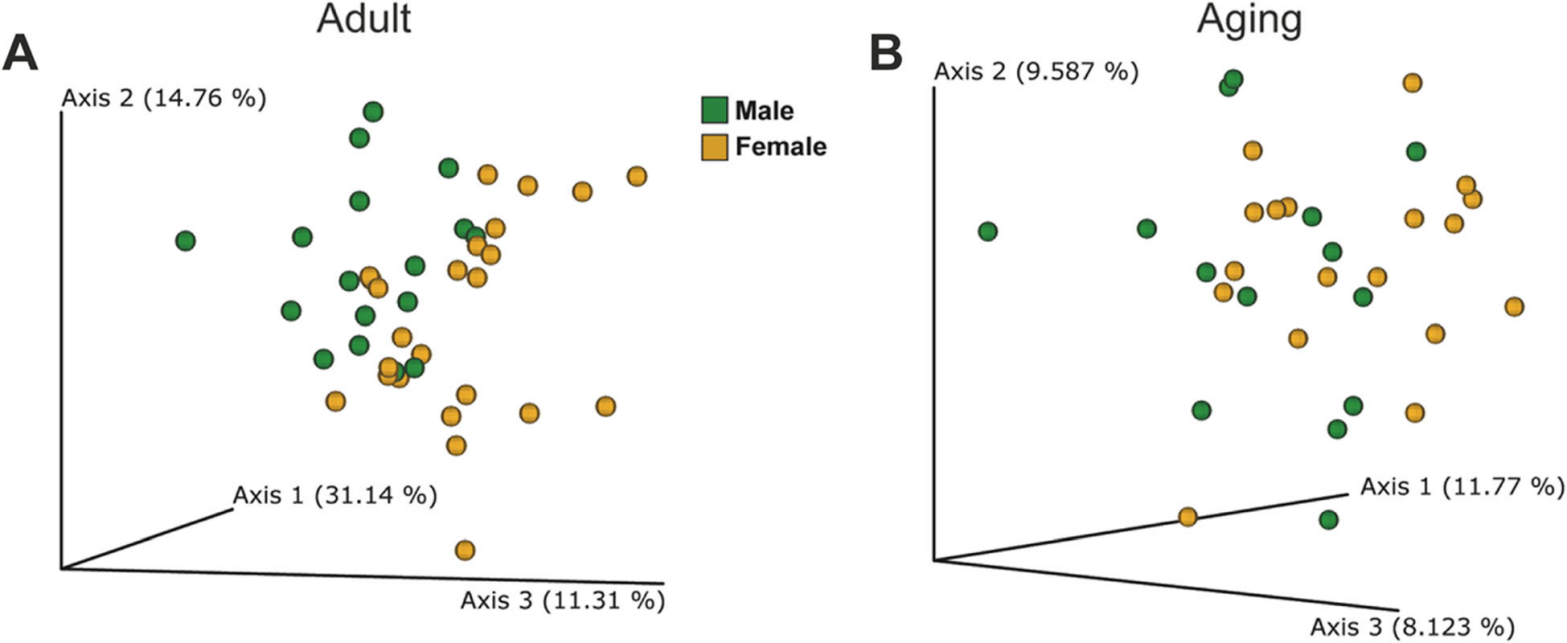
Microbial differences between male and female mice in the adult and aged mice groups. **a** Beta-diversity using Principal Coordinate Analysis (PCoA) of weighted UniFrac distances of male (*n* = 17) and female (*n* = 25) adult mice (*p* = 0.015) and **b** unweighted UniFrac distances of male (*n* = 14) and female (*n* = 18) aged mice (*p* = 0.015)

**Fig. 3:**
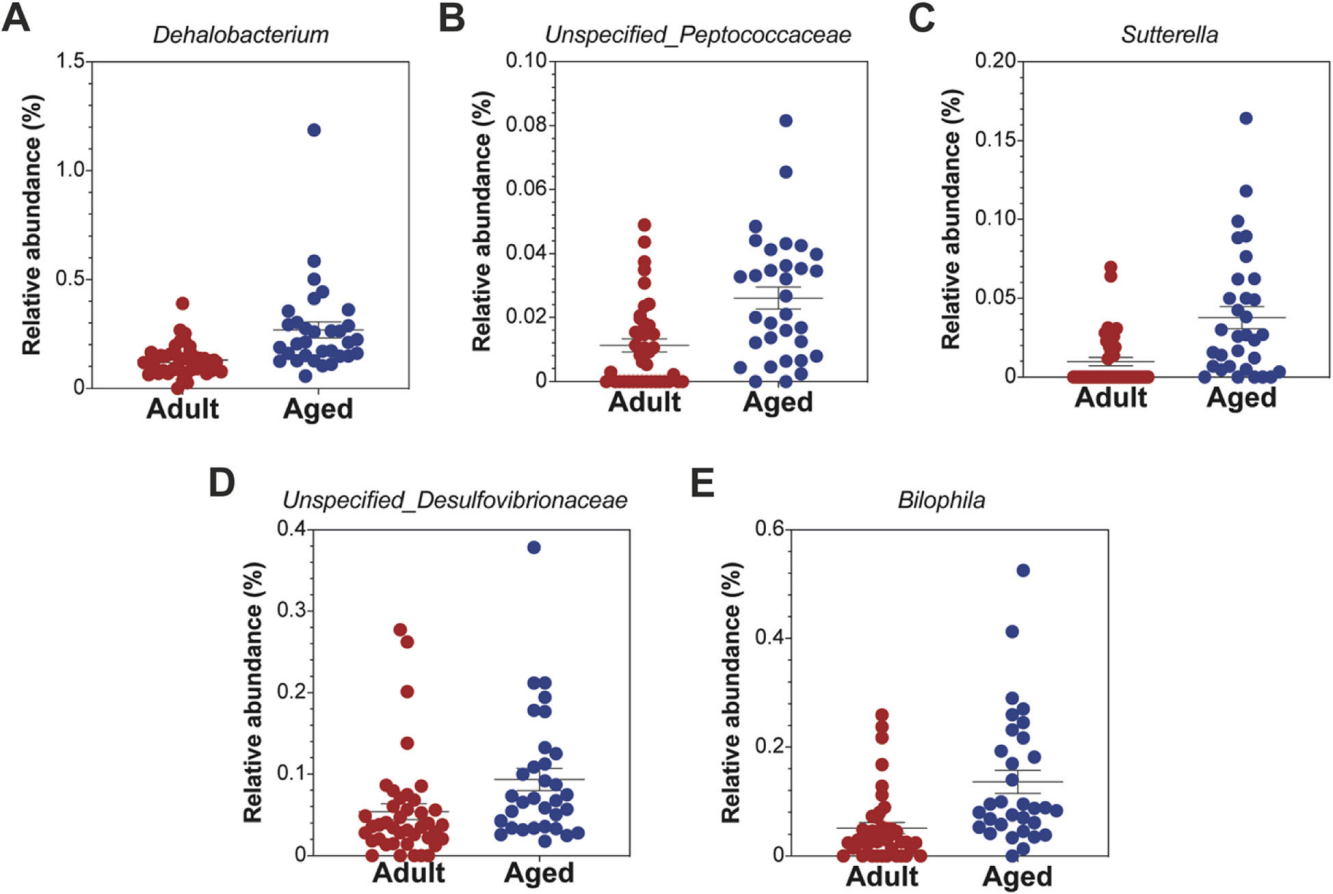
Genera that differed significantly between adult and aged mice. Analysis of microbiome composition (ANCOM) revealed 6 genera **a** *Dehalobacterium*, **b** unspecified *Peptococcaceae*, **c** *Sutterella*, **d** unspecified *Desulfovibrionaceae*, and **e** *Bilophila*, with significantly different relative abundance in adult versus aged mice

A comparison between males and females (in each group separately) revealed that the bacterial beta-diversity of males differed from that of females, in young and in adult mice (Fig. 2a, b). The taxa which differed significantly between the two groups regardless of gender are summarized in Fig. 3. The genera included *Dehalobacterium*, *Bilophila*, *Sutterella*, an unspecified *Desulfovibrionaceae*, and an unspecified *Peptococcaceae*, all of which were more abundant in the aged mice.

### Differences in metabolic parameters between adult and aged mice

**Fig. 4:**
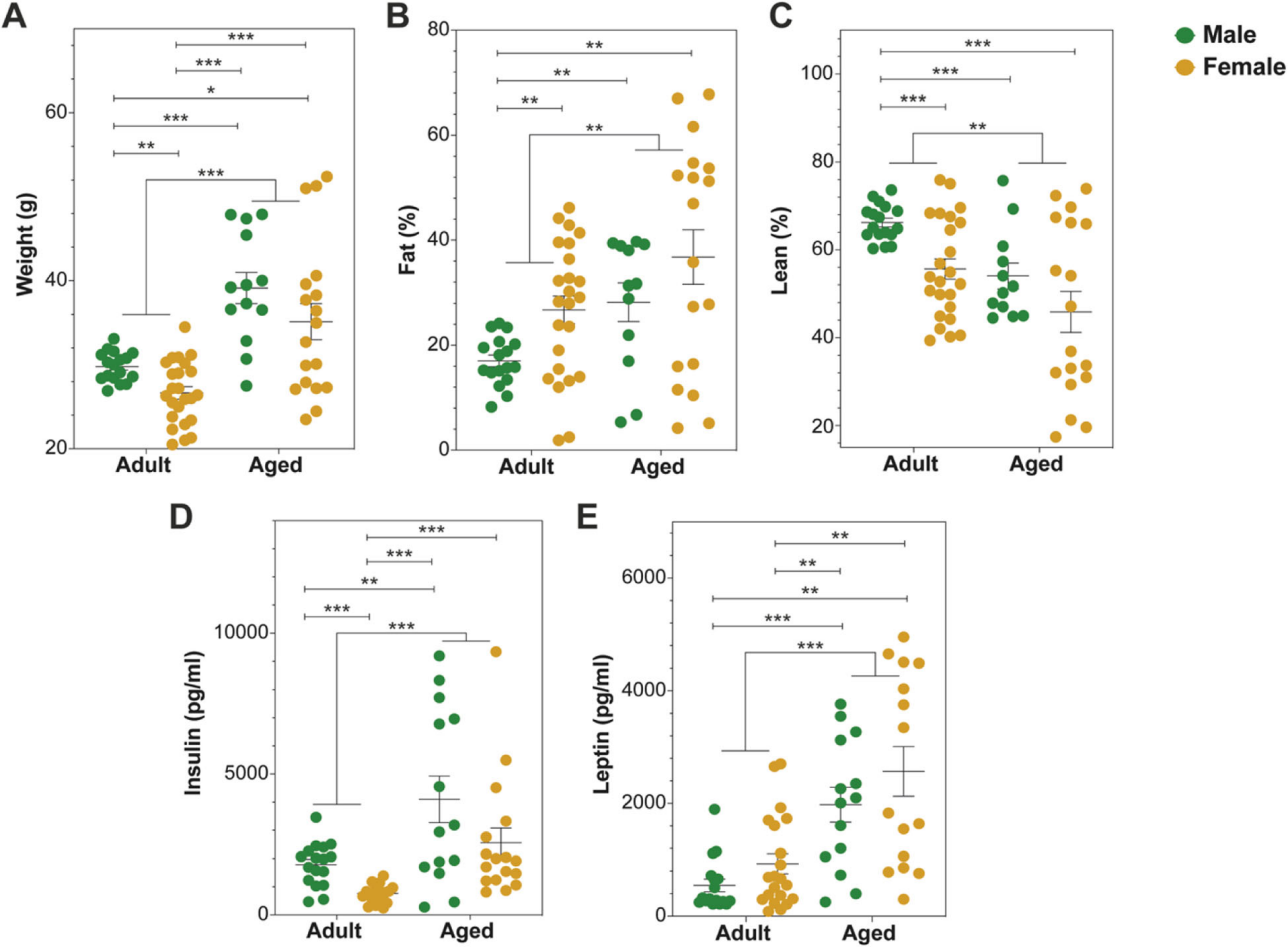
Comparison of weight, fat, lean body mass, insulin, and leptin measurements between adult and aged mice (separated by gender). Mice were weighed (**a**), and fat (**b**) and lean (**c**) mass were measured using MiniSpec NMR; insulin (**d**) and leptin (**e**) were determined from blood samples using Milliplex MAP multiplex assay. **p < 0.01 and ***p < 0.001

Using a body composition analyzer, we measured body weight and composition of adult and aged mice (separated by gender). Aged mice were significantly heavier (*p* < 0.0001, Fig. 4a), with increased fat (*p* = 0.0046, Fig. 4b) and reduced lean mass (*p* = 0.0014, Fig. 4c) compared to adult mice. In keeping with this, they were also hyperinsulinemic and hyperleptinemic (*p* < 0.0001 Fig. 4d and e, respectively). The gender effect was only seen in the adult mice where male mice differed from female mice in weight, fat (%), lean (%), and insulin levels. These gender-based differences were not seen in the aged mice (Fig. 4).

**Fig. 5:**
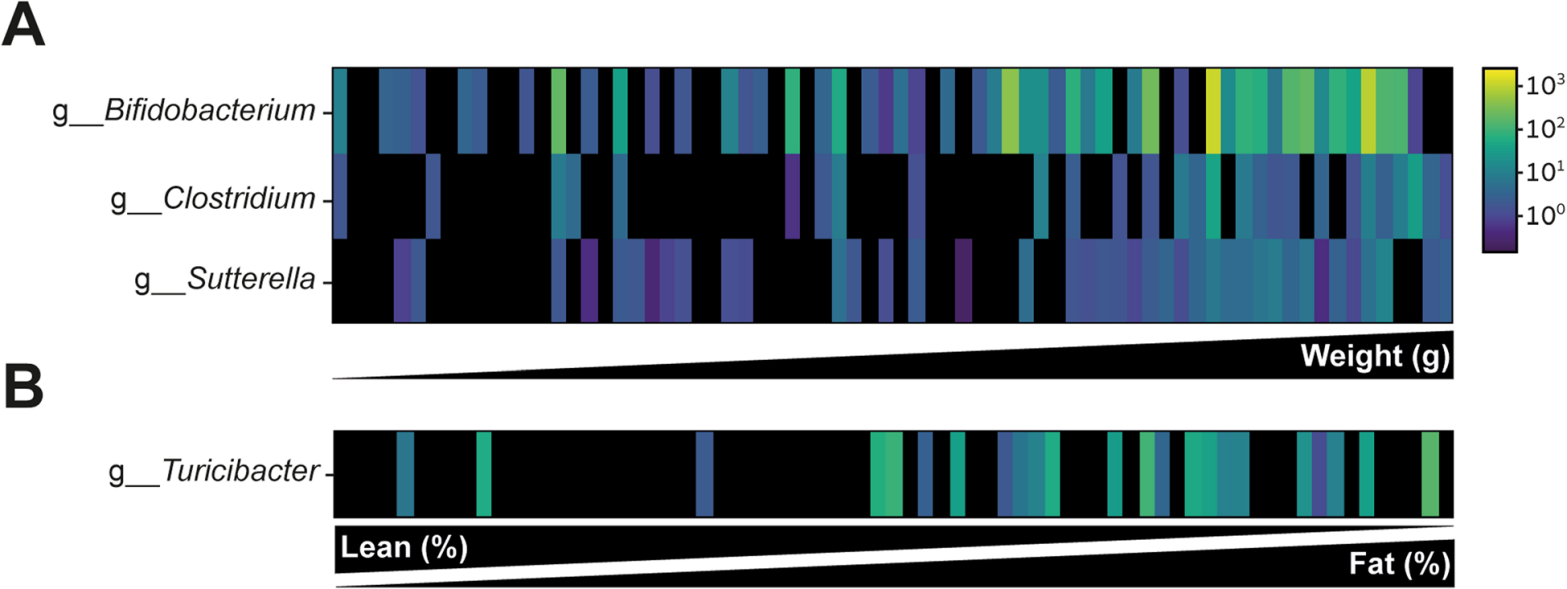
Bacteria that are associated with weight and fat/lean percent mass in both adult and aged mice. Taxa abundance is represented by color scale. **a** Three genera *Bifidobacterium*, *Clostridium*, and *Sutterella* were significantly correlated with weight. **b** One genus *Turicibacter* correlated with fat lean percent mass

We next looked which bacteria correlated with the tested parameters. *Bifidobacterium*, *Clostridium*, and *Sutterella* were positively correlated with higher weight (Fig. 5a) and the genus *Turicibacter* correlated with high fat mass (%) (Fig. 5b).

### The gut microbiota is responsible at least in part for the aged phenotype

**Fig. 6:**
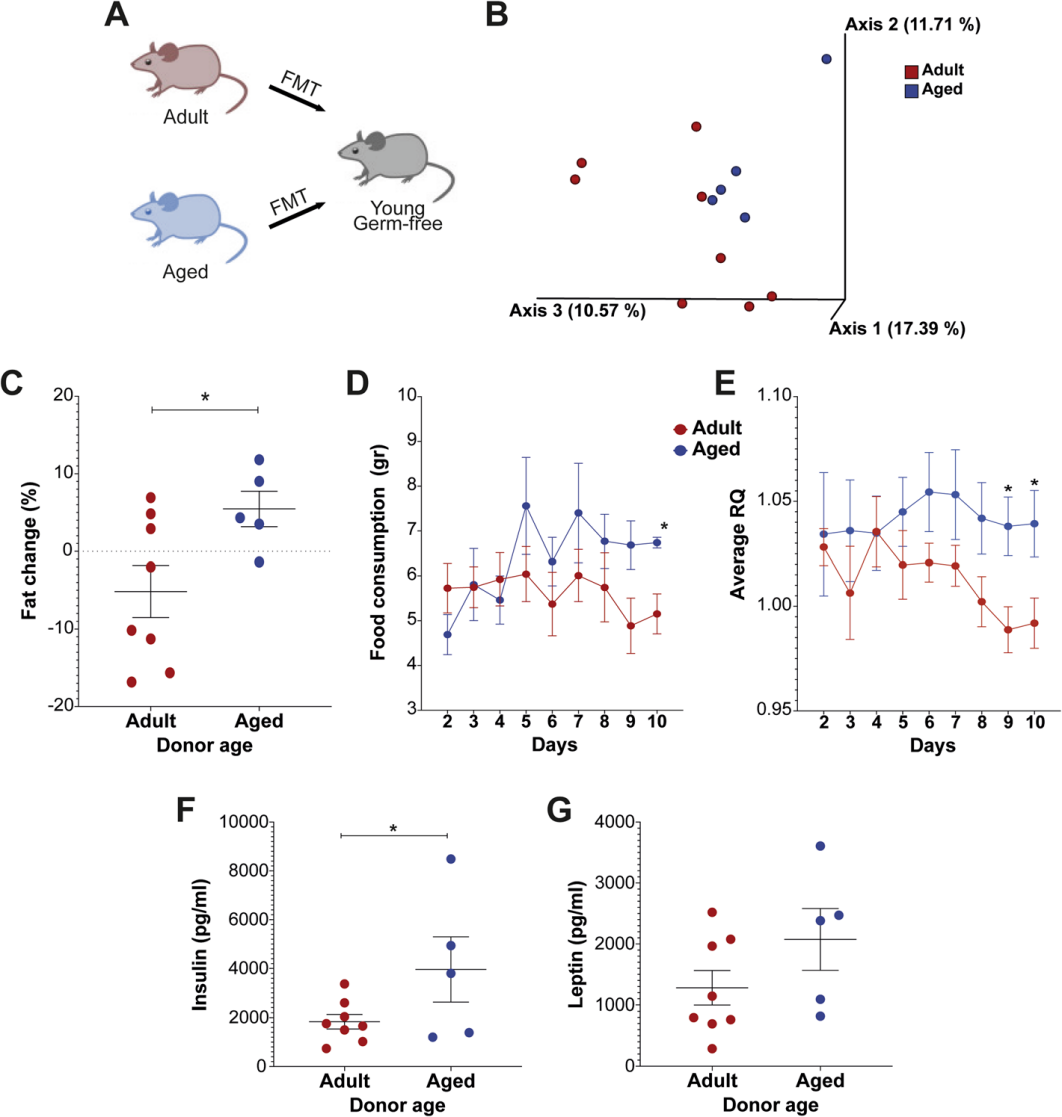
Changes in weight, fat, insulin, leptin and metabolic activity of germ-free mice receiving FMT from adult or aged mice. **a** Feces from adult (*n* = 8) and aging (*n* = 5) WT FVB/N mice were transplanted into 8-week-old germ-free mice through oral gavage. **b** Beta-diversity using Principal Coordinate Analysis (PCoA) of Jaccard distances (*p* = 0.057). Graphs show changes in the percentage of fat between days 0 and 10 days post transplant (**c**). Food consumption (**d**), and average respiratory quotient (RQ) (**e**) were measured from days 2–10. Insulin (**f**) and leptin (**g**) were measured in the blood on day 10. *p < 0.05, **p < 0.01, and ***p < 0.001

In order to examine the involvement of the gut microbiota in the metabolic parameters exhibited by aged mice, FMT was carried out. Feces were transplanted from adult and aged FVB/N mice into 8-week-old germ-free (GF) mice (Fig. 6a). The gut microbiome of GF mice who received adult mice feces differed from GF mice who received aged mice feces (*p* = 0.057, Fig. 6b). Both groups gained weight following transplantation, and no differences in weight gain were observed between the two groups. However, body fat mass gain was significantly higher in GF mice receiving FMT from aged mice, compared with GF mice receiving FMT from adult mice (*p* = 0.016, Fig. 6c). Mice transplanted from aged mice consumed more food towards the end of the experiment (*p* = 0.018 on day 10, Fig. 6d). The respiratory quotient (RQ) average was higher in GF mice receiving feces from aged mice versus those that received the FMT from adults (*p* = 0.018 on day 9 and *p* = 0.035 on day 10, Fig. 6e). Finally, higher levels of insulin (*p* = 0.039) and leptin (*p* = 0.082) were detected in the blood of GF mice receiving feces from aged versus adult mice (Fig. 6f, g), which is consistent with the changes seen in obesity.

Collectively, our findings show that adult and aged animals have a different gut microbiota and the gut microbiota is sufficient to transfer aged phenotypes to young GF recipients.

## Discussion

The aging process involves multiple factors and mechanisms. One of the many physiological parameters that change throughout aging is the gut microbiome [19]. In our study, we first examined the microbiome in adult and aged mice. We show that we can distinguish these groups based on their microbiome composition, a result that is consistent with previous studies that examined similar age groups [20, 21]. We also found a high Firmicutes to Bacteroidetes ratio in the aged group, which is associated with high-fat diet (“Western diet”), weight gain, and obesity [22, 23]. In addition we found several genera that were correlated with weight and have been described in association with obesity and weight gain phenotype [24–27]. *Bifidobacterium* has been positively correlated with Western-style diet [27]. Several species of *Clostridium* are associated with high-fat diet or obesity phenotypes [24–26] and *Sutterella* is associated with low-fat/high-sugar diet and was also shown to be elevated in aged mice (Fig. 2). The genus *Turicibacter* that was found in correlation with high fat percent and low lean percent mass was also implicated in obesity [28].

Indeed, we observed significantly higher weight and fat mass and lower lean mass in aged mice. A possible explanation might be that a decrease in basal metabolic rate and reduced physical activity that occurs during aging led to these changes as proposed by McMullan et al. [29]. In addition, serum insulin and leptin increased in aged mice. Hyperinsulinemia and hyperleptinemia are commonly associated with insulin or leptin resistance and type 2 diabetes [30–32]. These results indicate that aged mice have a gut microbiota that is associated with obesity and hyperinsulinemia and exhibit metabolic phenotypes related to obesity and hyperinsulinemia, as well.

After characterizing the gut microbiome and metabolic parameters in adult and aged mice in situ, we next examined the effect of the gut microbiome of these groups of mice on metabolic parameters via FMT to normal germ-free mice. The gut microbiome transmitted several of the phenotypes that we observed in aged mice. The fat mass increment was significantly higher in mice that received feces from aged mice. In addition, they consumed more food, had higher respiratory quotient (average RQ) and higher leptin and insulin levels compared to mice receiving adult feces. The FMT recipients also exhibited phenotypes characteristic of obesity. For instance, high RQ values have been associated with fat gain and indicate low fat and high carbohydrate oxidation [33–35]. High levels of leptin and insulin in the blood are associated with obesity [36, 37].

Based on such an association between aging and obesity, a condition that is also known to be a cause of mortality and a risk factor for several diseases [38], dietary modification may be helpful for maintaining the health of the aging population. Indeed, caloric restriction leads to longevity [39] and to alterations in the composition of the gut microbiome in mice [40]. In fact, in this study, we saw that the gut bacterial population itself is sufficient to induce some of the manifestations of obesity. If we can shift the bacterial population towards a healthier microbiome, we may be able to improve the quality of life of older populations.

## Conclusions

We conclude that aged mice have a gut microbiota with obesogenic characteristics. Our results show that the gut microbiome indeed transfers some of the obesity-related phenotypes from aged to young animals. Whether the opposite is true remains an open question. Should the answer be “yes,” this would definitely be a scenario to be further explored.

## Data Availability

The datasets generated during and analyzed during the current study are available in the European Nucleotide Archive repository with the accession number ERP119790 (https://www.ebi.ac.uk/ena/browser/view/PRJEB36583)[41].
